# The Neuroprotective Effect of Gugijihwang-Tang on Trimethyltin-Induced Memory Dysfunction in the Rat

**DOI:** 10.1155/2013/542081

**Published:** 2013-06-04

**Authors:** Eun-Yee Jung, Mi-Sook Lee, Chang Joon Ahn, Seung-Hun Cho, Hyunsu Bae, Insop Shim

**Affiliations:** ^1^Department of Basic Korean Medical Science, Acupuncture and Meridian Science Research Center, Kyung Hee University, Seoul 130-701, Republic of Korea; ^2^Hospital of Korean Medicine, College of Korean Medicine, Kyung Hee University, Seoul 130-701, Republic of Korea; ^3^Department of Basic Oriental Medical Science, College of Oriental Medicine, Kyung Hee University, Seoul 130-701, Republic of Korea

## Abstract

Gugijihwang-Tang (the herbal formula PM012), a decoction consisting of several herbs including *Rehmanniae Radix Preparata*, has been widely used as herbal treatment for dementia. In order to investigate the neuroprotective action of this prescription, we examined the effect of Gugijihwang-Tang on learning and memory using the Morris water maze and [F-18]FDG micro PET neuroimaging technique. After injection of trimethyltin (TMT, 8.0 mg/kg, i.p.), which is a potent toxicant that selectively kills cells in the central nervous system, rats were administered Gugijihwang-Tang (100 mg/kg, p.o.) daily for two weeks, followed by the Morris water maze tasks and [F-18]FDG micro PET neuroimaging. In Gugijihwang-Tang administered TMT-treated rats, they showed improved learning and memory abilities in water maze tasks and glucose metabolism, suggesting that Gugijihwang-Tang plays effectively positive role in the improvement of brain function including learning and memory after TMT-induced neurodegeneration. Taken together, our results suggested that the Gugijihwang-Tang should be useful for developing strategies protecting nervous system and improving brain function.

## 1. Introduction

Dementia is a brain disorder that seriously affects a person's ability to carry out daily activities and its causes generally include primary neurodegenerative disorders, with the most prevalent being Alzheimer's disease (AD) [1]. Trimethyltin (TMT) is an organotoxic compound with potent neurotoxicant effects which is regarded as being particularly useful for studying the response to injury on account of the distinct pattern of neuronal degeneration in rodent brain. When rat is treated with TMT, it causes a loss of pyramidal neurons predominantly in the rat hippocampal CA3 region starting 3-4 days later [2–4]. Behaviorally, TMT intoxication is characterized by seizure, self-biting, aggressive behavior, hyperactivity, and impairment of working memory [5–7]. The precise mechanism of selective and delayed neuronal death after TMT administration has not been clarified yet. Intoxication with TMT leads to profound neurodegeneration, behavioral and cognitive deficits in both humans and experimental animals [6]. In rats, TMT induced the loss of pyramidal neurons in the hippocampus at 4 days, which was robust at 14 days after TMT administration [8]. These anatomical and behavioral findings have made TMT-intoxicated rats an attractive model for degenerative diseases such as AD, the most common cause of dementia [9, 10]. Herbal medicines, such as *Ginkgo biloba, Ginseng*, or *Melissa officinalis*, have been commonly used as memory or cognition enhancers. The effects of these enhancers have been demonstrated scientifically [11, 12]. *Yukmi-jiwang gamibang* (YJG) has a significant effect on memory enhancement and the expression of genes associated with not only the prevention of neuronal degeneration but also neuronal growth events [13]. Recent double-blind, placebo-controlled trials have also demonstrated that *Yukmi-jiwang gamibang* significantly enhances cognitive abilities in normal human subjects [14]. However, there have been few reported studies on accessing learning and memory enhancement triggered by treatment with YJG in dementia animal models. Gugijihwang-Tang (GJT), the herbal formula PM012, is another memory or cognition enhancer which is composed of 7 herbal medicines, including steamed *Rehmanniac radix Preparata*, *Lycium chinense, Dioscoreae radix, Corni fructus, Hoelen, Mountain cortex radix, and Alismatis radix*. GJT is composed of 7 herbal medicines which are 6 herbal medicines of *Yukmi-jiwang gamibang* and *Lycium chinense*. Thus, we examined the effect of GJT (YJG and *Lycium chinense*) on learning and memory ability in TMT-induced amnesia rats using the Morris water maze. Furthermore, we examined brain activity using the technique of positron emission tomography (PET) with ^18^F-2 fluoro-2-deoxy-D-glucose ([F-18]FDG) that allows autoradiographic assessment of radiolabeled 2-deoxyglucose uptake. [F-18]FDG micro PET scan, a functional neuroimaging technique, PET brain imaging has been used for the diagnosis and assessment of cerebrovascular diseases, such as dementia. More recently, [F-18]FDG micro PET scan became available for visualizing brain activity in small rodents [15, 16]. We hypothesized applying this technique in TMT-induced dementia animal model and memory improving substance (GJT). Thus, we have attempted to measure brain activity changes with the technique in TMT-treated rats.

## 2. Materials and Method

### 2.1. Animals

Male Sprague-Dawley rats weighting 250–280 g each were purchased from Samtaco Animal Corp. (Kyungki-do, Republic of Korea). The animals were allowed to acclimatize themselves for at least 7 days prior to the experimentation. The animals were housed in individual cages under light-controlled conditions (12/12 hr light/dark cycle) and at 23°C room temperature. Food and water were available ad libitum.

### 2.2. Preparation of Gugijihwang-Tang (GJT)

The contents and the ratio of each component in Gugijihwang-Tang are shown in Table 1.

### 2.3. Reagents

Trimethyltin chloride (TMT; Sigma-Aldrich Inc., St. Louis, MO, USA) or 0.9% saline (vehicle) was injected and Gugijihwang-Tang was administered into rats during 2 weeks.

### 2.4. Experimental Design

Rats were randomly assigned into three groups (*n* = 10 per group): the normal group (vehicle group), the TMT-lesioned + saline group (the control group), and TMT-lesioned + Gugijihwang-Tang (100 mg/kg treated animals, the GJT group). The rats were injected intraperitoneally (i.p.) with TMT (8.0 mg/kg, body weight) dissolved in 0.9% saline and then returned to their home cages. Gugijihwang-Tang (100 mg/kg, p.o.) was dissolved in saline and orally administered for two weeks after TMT-induced neurodegeneration. To verify that TMT causes damage to hippocampal neurons, Nissl staining was performed using a previously described procedure (see Supplementary Figure  1 in the Supplementary Material available online at http://dx.doi.org/10.1155/2013/542081) [17].

The water maze test was performed for one week and [F-18] FDG micro PET neuroimaging from the 15th day after the injection of TMT.

### 2.5. Water Maze Test

The swimming pool of the Morris water maze was a circular water tank 200 cm in diameter and 35 cm deep. It was filled to a depth of 21 cm with water at 23 ± 2°C. A platform 15 cm in diameter and 20 cm in height was placed inside the tank with its top surface being 1.5 cm below the surface of the water. The pool was surrounded by many cues that were external to the maze. A CCD camera was equipped with a personal computer for the behavioral analysis. Each rat received four daily trials. For 6 consecutive days, the rats were tested with three acquisition tests. They also received retention tests on the 7th day. For the acquisition test, the rat was allowed to search for the hidden platform for 180 s, and the latency to escape onto the platform was recorded. The animals were trained to find the platform that was in a fixed position during 6 days for the acquisition test, and, then for the retention test, they received a 1 min probe trial in which the platform was removed from the pool. The interval time was 1 min. Performance of the test animals in each water maze trial was assessed by a personal computer for the behavioral analysis (S-mart program, Spain).

### 2.6. [F-18]FDG Micro PET Scan

All rats were deprived of food for 12–15 h before the experiments to enhance [F-18]FDG uptake in the brain. Each animal was placed on a heating pad in a cage and warmed for at least 30 min before the [F-18]FDG injection. The temperature of the cages was kept at 30°C throughout the uptake period in accordance with an optimized [F-18]FDG uptake protocol [18]. [F-18]FDG (500 *μ*Ci/100 g body weight) was injected through tail vein. After [F-18]FDG injection, radioactivity in rat brain tissue initially rises rapidly. During [F-18]FDG micro PET scans, the animals were kept under isoflurane inhalation anesthesia (2% in 100% oxygen; IsoFlo; Abbott Laboratories).

### 2.7. Voxel-Based Statistical Analysis

Voxel-based statistical analysis was performed to compare the cerebral glucose metabolism of the group's datasets. The procedure used for SPM analysis of the animal PET data was as previously described in our previous study [18]. Briefly, for efficient spatial normalization, only the brain region was extracted. A study-specific template was then constructed using all the datasets. The PET data was spatially normalized onto a rat brain template and smoothed using a 3 mm Gaussian kernel. Count normalization was performed. A voxelwise *t*-test between the group's datasets was performed using the Statistical Parametric Mapping 5 program (*P* < 0.05, *K* > 50).

### 2.8. Behavioral Data Analysis

The data were presented as means ± S.E.M. Behavioral data (acquisition and retention data) were statistically analyzed by ANOVA testing with repeated measures on the time factor. The origin of the significant effects was further examined by posthoc comparisons using the LSD technique. The criterion for statistical significance was considered to be *P* values <0.05.

## 3. Results

### 3.1. Effect of Gugijihwang-Tang on Performance in Water Maze Task

**Figure 1:**
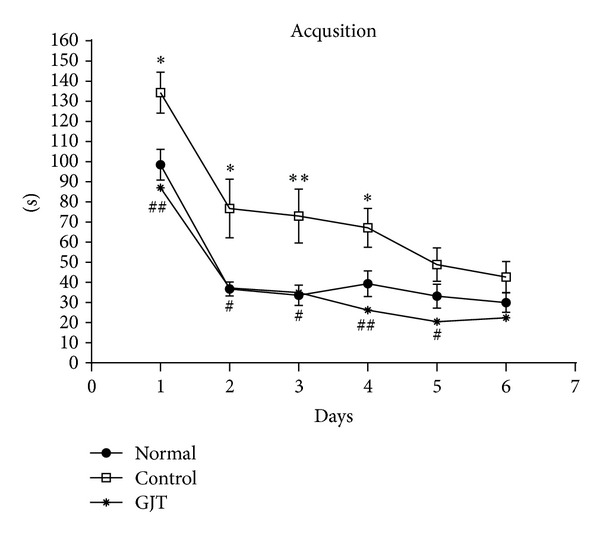
The latency to escape onto the hidden platform during the Morris water maze. The task was performed with 3 trials per day during 6 days for the acquisition test. The values are presented as means ± S.E.M. *P < 0.05, **P < 0.01, compared with the normal group; ^#^ *P* < 0.05, ^##^ *P* < 0.01, compared with the control group.

The latency to escape onto the hidden platform during the acquisition trial of the water maze was recorded, and the results are depicted in Figure 1. The escape latency differed among the groups when the results were averaged over all the session. The latency to find the hidden platform of the normal and GJT groups was significantly decreased compared with the control group. TMT severely impaired spatial cognition in the water maze test, and the GJT group ameliorated TMT-induced learning and memory deficits in the water maze.

**Figure 2:**
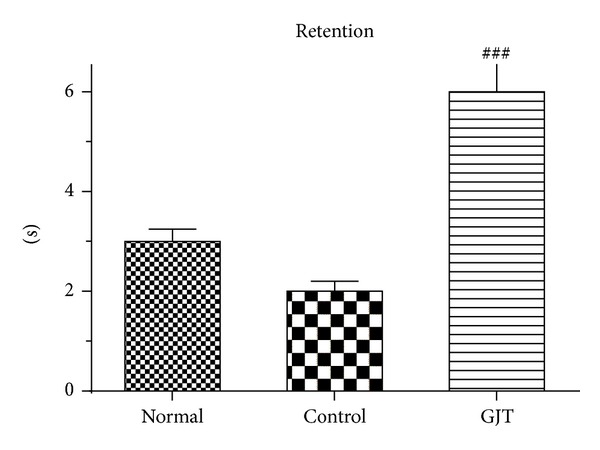
Retention performance was tested on the 7th day. The rats received a 1 min probe trial in which the platform was removed from the pool for retention testing. The values are presented as means ± S.E.M., ^###^ *P* < 0.001, compared with the control group.

The total time spent on the platform was used to evaluate the spatial performance of the rat during retention trials. The results of the retention test at the 7th day are depicted in Figure 2. The time spent around platform among the groups differed. The time spent around the platform was 3.3 ± 0.84 in the normal group, 2.3 ± 0.53 in the control group, and 6.2 ± 2.2 in the GJT group. The normal and GJT groups spent more time around the platform than did the control group. The GJT group showed a significant increase (*P* < 0.001) in retention time compared to the control group (Figure 2).

### 3.2. [F-18]FDG Micro PET Neuroimaging in the Brain Changes in the Whole Brain Glucose Metabolism in TMT-Induced Neurodegeneration and Treatment of GJT

**Figure 3:**
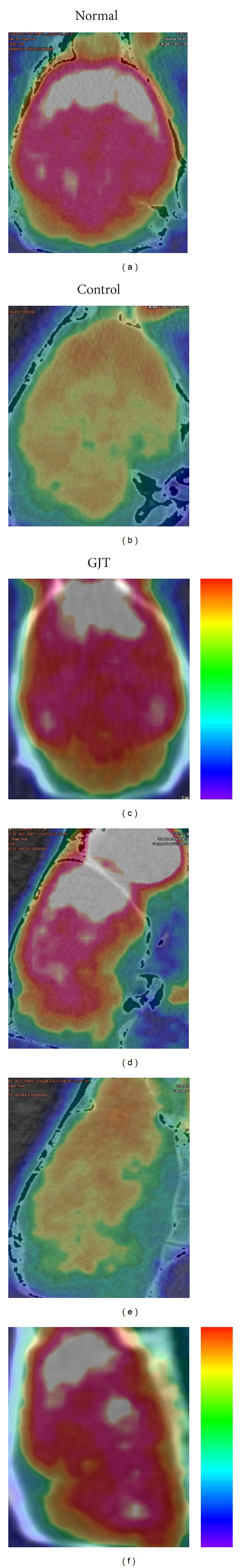
Changes in whole brain glucose metabolism in TMT-induced neurogeneration and treatment of GJT. (a and d) Normal, (b and e) control, and (c and f) GJT groups, (a to c) axial and (d to f) saggital section of the brain.

**Figure 4:**
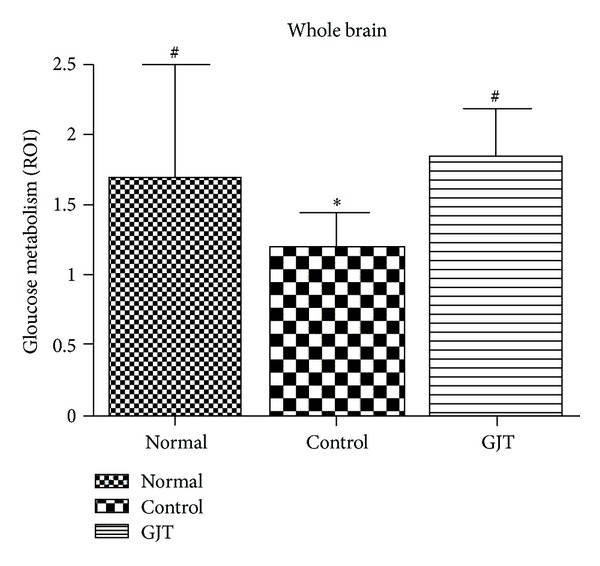
Changes in whole brain glucose metabolism in TMT-induced neurogeneration and treatment of GJT. The values are presented as means ± S.E.M. *P < 0.05, compared with the normal group, ^#^ *P* < 0.05, compared with the control group.

Figure 3 shows changes in glucose metabolism of each group in axial and saggital section of the whole brain.  Figure 4 summarizes these changes in graph indicating statistical difference between groups.

**Figure 5:**
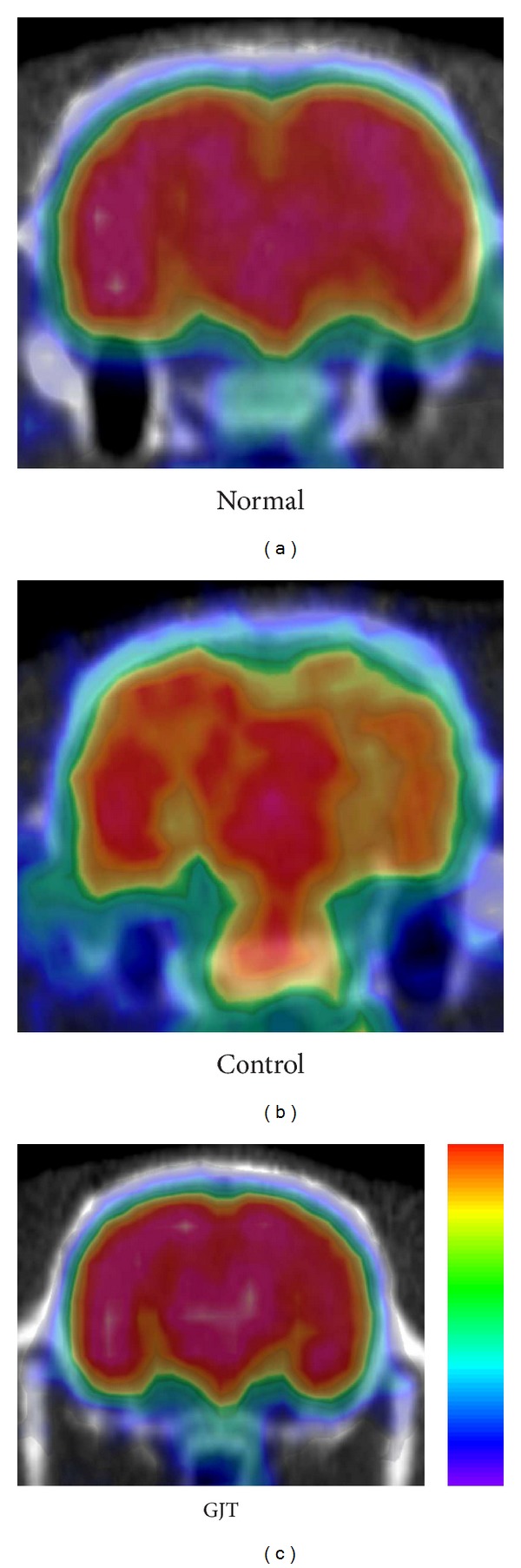
Changes in the region of the hippocampus glucose metabolism in TMT-induced neurodegeneration and treatment of GJT. (a to c) Coronal section of the brain.

**Figure 6:**
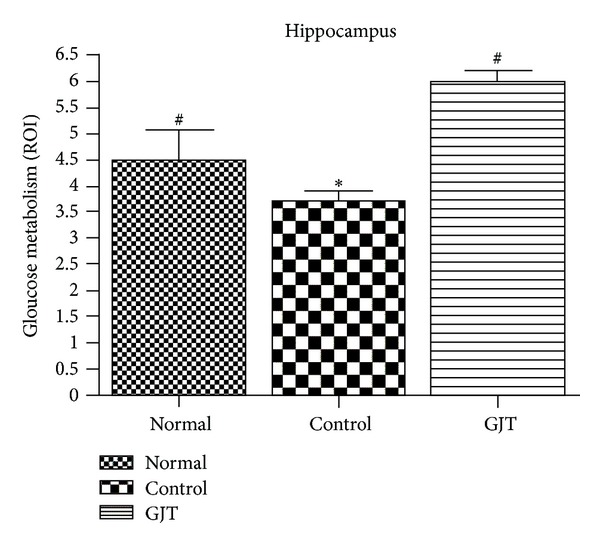
Changes in the region of the hippocampus glucose metabolism in TMT-induced neurodegeneration and treatment of GJT. (a to c) Coronal section of the brain. The value are presented as means ± S.E.M. *P < 0.05, compared with the normal group, ^#^ *P* < 0.05, compared with the control group.

Figure 5 shows changes in glucose metabolism of each group in coronal section of the region of the hippocampus.  Figure 6 summarizes these changes in graph indicating statistical difference between groups. The control group significantly reduced glucose metabolism, exhibiting a 19% decrease in [F-18]FDG uptake in the whole brain (*P* < 0.05) and a 30% decrease in the hippocampus (*P* < 0.05) when compared to the normal group. Treatment with GJT can significantly reverse hypometabolism as shown by increasing [F-18]FDG uptake by 67% in the whole brain (*P* < 0.05) and 62% in the hippocampus (*P* < 0.05).

## 4. Discussion

Several studies have demonstrated behavioral abnormalities such as increased seizure susceptibility, aggression, and learning impairment after TMT-induced neurodegeneration, which is consistent with results of this study [5, 19]. The present study confirms previous findings that TMT treatment induces learning impairment from Morris water maze test. This study thus strongly proposes TMT-treated rat to be a good model for studying neuronal degeneration. This model may be also useful for elucidating mechanisms underlying resistance to dementia and excite toxic injury in the hippocampus. Other many studies clearly exhibit the functional significance of the hippocampal neurodegeneration by TMT [2, 5, 19, 20]. The hippocampus possesses information derived from the associated brain regions that are involved in learning, memory, emotion, and motivation [20]. In TMT exposed rats, GJT treatment improved spatial learning and memory in Morris water maze, suggesting that GJT can reduce spatial deficits on the central nervous system after TMT-induced neurodegeneration. The latency of the GJT group to find the hidden platform was significantly decreased on the acquisition testing, suggesting that GJT could improve the acquisition and retention deficits on the Morris water maze testing. Although the precise mechanisms for recovery of such behavioral deficits following the administering of GJT are not clear, this present study collectively demonstrates that GJT ameliorated learning and memory deficits through its neuroprotective effects on the central nervous system, and thus may have beneficial effects on TMT-induced cognitive impairment.

Numerous studies from epidemiological [21, 22] and functional neuroimaging [23] studies indicate that the dysregulation of energy metabolism in the brain is an important causative factor in the developing dementia. PET imaging has been used for the assessment of dementia and can be used in the diagnosis of disease and new therapeutic searching. More recently, [F-18]FDG micro PET scan became available for visualizing brain activity in small rodents [15, 16]. [F-18]FDG micro PET neuroimaging effects from those components in GJT may have been responsible for the protection against TMT-induced neurodegeneration and cognitive deficits shown in the present study. Thus herbal medicine therapy using GJT in the TMT-induced neurodegeneration brain may have a solid therapeutic potential as a treatment for dementia and amnesia.

## Supplementary Material

Supplemental Figure 1: Histological findings for the hippocampus after TMT treatment. Representative images of a Nissl-stained histological section. (A) No unusual histological findings were observed in vehicle-treated controls. (B) The number of loss of pyramidal neurons was seen in the CA3 region (arrow) 14 days after TMT treatment.

## Figures and Tables

**Table 1:** The contents of Gugijihwang-Tang and the amounts of standard materials.

Herbal medicines	Ratio (%)
*Rehmanniae radix Preparant *	8 (28.6%)
*Cornii fructus *	4 (14.3%)
*Lycium chinense *	3 (10.7%)
*Discoreae radix *	4 (14.3%)
*Hoelen *	3 (10.7%)
*Mountain cortex radix *	3 (10.7%)
*Alismatis radix *	3 (10.7%)
