# Supplementary material for: The Neuroprotective Effect of Gugijihwang-Tang on Trimethyltin-Induced Memory Dysfunction in the Rat
Source: Evid Based Complement Alternat Med. 2013 Jun 4;2013:542081. doi: 10.1155/2013/542081 (PMC3687724; doi:10.1155/2013/542081)

**Supplementary Fig.1.** Histological findings for the hippocampus after TMT treatment. Representative images of a Nissl-stained histological section. (A) No unusual histological findings were observed in vehicle-treated controls. (B) The number of loss of pyramidal neurons was seen in the CA3 region (arrow) 14 days after TMT treatment. Scale bars in A represent 200 μm.

Supplementary Fig.1


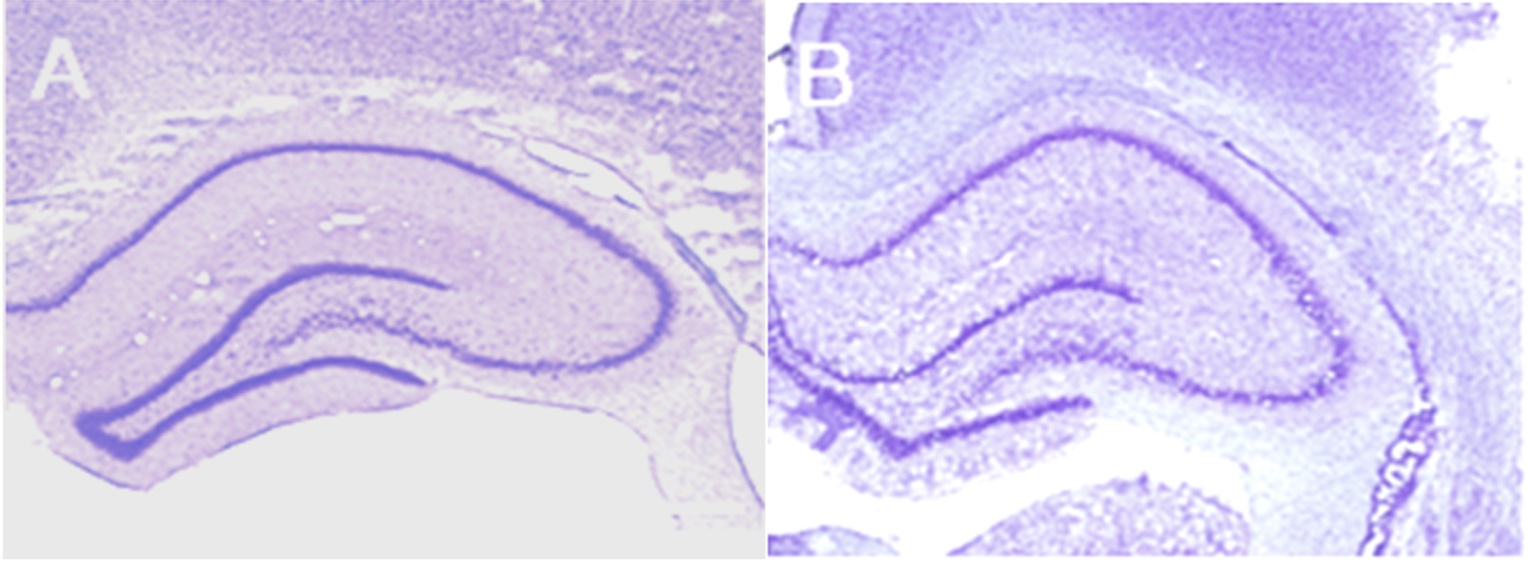

Supplement: Supplementary file 1 — Supplemental Figure 1: Histological findings for the hippocampus after TMT treatment. Representative images of a Nissl-stained histological section. (A) No unusual histological findings were observed in vehicle-treated controls. (B) The number of loss of pyramidal neurons was seen in the CA3 region (arrow) 14 days after TMT treatment. [file 542081.f1.docx]
